# Auditory Dysfunction Among Individuals With Neurofibromatosis Type 1

**DOI:** 10.1001/jamanetworkopen.2021.36842

**Published:** 2021-12-06

**Authors:** Gary Rance, Julien Zanin, Alice Maier, Donella Chisari, Kristina M. Haebich, Kathryn N. North, Gabriel Dabscheck, Marc L. Seal, Martin B. Delatycki, Jonathan M. Payne

**Affiliations:** 1 Department of Audiology and Speech Pathology, The University of Melbourne, Carlton, Victoria, Australia; 2 Murdoch Children’s Research Institute, Parkville, Victoria, Australia; 3 Department of Paediatrics, Faculty of Medicine, Dentistry and Health Sciences, University of Melbourne, Parkville, Victoria, Australia; 4 Victorian Clinical Genetics Services, The Royal Children’s Hospital, Parkville, Victoria, Australia; 5 The Royal Children’s Hospital, Parkville, Victoria, Melbourne

## Abstract

**Importance:**

Neurofibromatosis type 1 (NF1) affects hearing through disruption of central auditory processing. The mechanisms, functional severity, and management implications are unclear.

**Objective:**

To investigate auditory neural dysfunction and its perceptual consequences in individuals with NF1.

**Design, Setting, and Participants:**

This case-control study included children and adults with NF1 and control participants matched on age, sex, and hearing level. Patients were recruited through specialist neurofibromatosis and neurogenetic outpatient clinics between April and September 2019. An evaluation of auditory neural activity, monaural/binaural processing, and functional hearing was conducted. Diffusion-weighted magnetic resonance imaging (MRI) data were collected from a subset of participants (10 children with NF1 and 10 matched control participants) and evaluated using a fixel-based analysis of apparent fiber density.

**Main Outcomes and Measures:**

Type and severity of auditory dysfunction evaluated via laboratory testing and questionnaire data.

**Results:**

A total of 44 participants (18 [41%] female individuals) with NF1 with a mean (SD) age of 16.9 (10.7) years and 44 control participants (18 [41%] female individuals) with a mean (SD) age of 17.2 (10.2) years were included in the study. Overall, 11 participants (25%) with NF1 presented with evidence of auditory neural dysfunction, including absent, delayed, or low amplitude electrophysiological responses from the auditory nerve and/or brainstem, compared with 1 participant (2%) in the control group (odds ratio [OR], 13.03; 95% CI, 1.59-106.95). Furthermore, 14 participants (32%) with NF1 showed clinically abnormal speech perception in background noise compared with 1 participant (2%) in the control group (OR, 20.07; 95% CI, 2.50-160.89). Analysis of diffusion-weighted MRI data of participants with NF1 showed significantly lower apparent fiber density within the ascending auditory brainstem pathways. The regions identified corresponded to the neural dysfunction measured using electrophysiological assessment.

**Conclusions and Relevance:**

The findings of this case-control study could represent new neurobiological and clinical features of NF1. Auditory dysfunction severe enough to impede developmental progress in children and restrict communication in older participants is a common neurobiological feature of the disorder.

## Key Points

**Question:**

Is neurofibromatosis type 1 (NF1) associated with auditory neural dysfunction and functional hearing consequences?

**Findings:**

In this case-control study of 44 individuals with NF1 and 44 matched control participants, 1 in 4 participants with NF1 showed evidence of auditory neural dysfunction, and 1 in 3 presented with abnormal functional hearing (speech perception). Diffusion-weighted magnetic resonance imaging analysis showed that participants with NF1 had significantly lower apparent fiber density within the ascending tracts of the brainstem.

**Meaning:**

The findings of this study suggest that auditory dysfunction severe enough to impede developmental progress in children and restrict communication in older participants is a common neurobiological feature of NF1.

## Teaser

This cohort study investigates auditory neural dysfunction and its perceptual consequences in individuals with neurofibromatosis type 1.

## Introduction

Neurofibromatosis type 1 (NF1) is an autosomal dominant condition with a birth incidence of approximately 1 in 2700 and is caused by loss-of-function alterations within the *NF1* gene (OMIM 613113).^1^ Although characterized by diverse cutaneous, skeletal, and neoplastic manifestations, cognitive deficits and behavioral problems are also common.^2,3^ Intelligence typically falls within the low to average range, and as many as 80% of children with NF1 experience executive dysfunction,^4^ inattention,^5^ and visuoperception deficits.^2^

Little is known about the hearing of patients with NF1. Most affected individuals show normal sound detection thresholds, but abnormal results on auditory neural and temporal processing tests have been reported.^6,7^ As such, central processing deficits may be a contributing factor in the high prevalence of speech and/or communication delays in NF1 populations.^8,9^

Disruption of firing patterns in the auditory nerve and brainstem affects perception. This has been most thoroughly investigated in patients with auditory neuropathy, who present with normal cochlear hair-cell function but disrupted electrophysiological potentials from the auditory nerve and central pathways.^10,11^ Functional consequences of auditory neuropathy include reduced capacity to perceive the timing cues that differentiate speech sounds,^12,13^ impaired ability to localize sound sources,^14^ and inability to understand speech in background noise.^15^

In the current study, we used a case-control design to investigate auditory neural function, monaural/binaural processing, and functional hearing in patients with NF1. In a subset of pediatric participants, we then used diffusion-weighted magnetic resonance imaging (dMRI) data to determine whether any regions in the auditory brainstem pathways show microstructural differences in patients with NF1 compared with control participants.

## Methods

### Participants

Informed consent was obtained from all parents or guardians, and age-appropriate assent was obtained prior to participation. Protocols were approved by the ethics committees of the Eye and Ear and Royal Children’s Hospitals, Melbourne. This study followed the Strengthening the Reporting of Observational Studies in Epidemiology (STROBE) reporting guideline.

Forty-four participants with NF1 were recruited from the Royal Children’s Hospital, Neurofibromatosis Clinic between April and December 2019. Group demographic characteristics are shown in Table 1. All were diagnosed by an expert neurologist or clinical geneticist based on clinical criteria.^16^ Study exclusion criteria were (1) participant or parent/guardian not fluent in English; (2) symptomatic intracranial pathology, including acquired brain injury or progressive intracranial tumor (asymptomatic/untreated lesions, such as optic gliomas, were eligible); (3) known intellectual disability (ie, IQ <70).^17^ Forty-four control participants individually matched for age (±12 months), sex, and hearing level (±5 dB) were also recruited to the study.

**Table 1.** Participant Demographic Characteristics

Group	No.	Female participants, No. (%)	Male participants, No. (%)	Mean (SD) [range]	
				Age, y	Hearing level, dBHL
Audiologic study					
NF1	44	18 (41)	26 (59)	16.9 (10.7) [6 to 56]	15.5 (5.8) [0.0 to 38.7]
Control	44	18 (41)	26 (59)	17.2 (10.2) [6 to 53]	13.2 (5.7) [−2.5 to 40.0]
MRI substudy					
NF1	10	8 (80)	2 (20)	11.5 (2.1) [9 to 15]	13.9 (3.3) [8.8 to 16.3]
Control	10	8 (80)	2 (20)	12.4 (2.1) [8 to 15]	13.4 (2.3) [10 to 14.4]

Abbreviations: dBHL, dB hearing level; MRI, magnetic resonance imaging; NF1, neurofibromatosis type 1.

### Auditory Phenotype

#### Sound Detection Levels

Behavioral audiometric thresholds were established to pure-tone stimuli at octave frequencies between 250 Hz and 8 kHz. Each ear was tested separately, and the ear with poorer detection thresholds was used for subsequent (monaural) assessments.

#### Electrophysiology

Auditory brainstem responses (ABRs) were recorded to acoustic clicks presented at 90 dB normed for a 100-microsecond stimulus (dBnHL). Responses were obtained to stimuli at presentation rates of 8, 33, 57, 75, and 100 Hz. Electroencephalographic samples following 2000 stimuli were averaged to produce each test run. Two runs were obtained and compared to determine waveform repeatability. Analysis was carried out independently by 2 experienced clinicians (G.R. and J.Z.) blinded to group status. These judges determined response presence/absence; poststimulus latency of waves I, III, and V; and peak-to-peak amplitude of waves I and V for stimuli presented at 33 Hz.

#### Auditory Temporal Processing

Auditory temporal resolution was assessed using an amplitude modulation detection task. The psychophysical protocol used an adaptive, 3-alternative task that sought the minimum detectable depth of sinusoidal modulation at 10 Hz and 150 Hz.^18^

#### Speech Perception

Binaural speech perception in background noise assessment was carried out using the Listening in Spatialized Noise (LiSN-S) test, which measures the participant’s ability to segregate a target speech signal from competing speech noise.^19^ The test was administered using headphones. A 3-dimensional auditory environment was created by synthesizing the test stimuli using a head-related transfer function. Participants were presented with a series of 20 to 30 target sentences and scored on the number of words in each sentence they accurately identified. Speech reception threshold (SRT) was established by varying the level of the stimulus sentences relative to the background noise to determine the signal-to-noise ratio required to identify 50% of the target words. The SRT was established in 4 conditions that varied in terms of noise location (0° vs 90° azimuth) and vocal quality of speaker used to produce the target and background signals (same or different voice). The 4 conditions were DV90 (different voices spatially separated by 90°); SV90 (same voice separated by 90°); DV0 (different voices from same direction); and SV0 (same voice from same direction).

#### Self-reported Hearing Disability

The Speech, Spatial, and Qualities of Hearing Scale (SSQ) was used to assess participants’ perceived hearing abilities across a variety of listening scenarios.^20^ Three listening domains were evaluated: speech understanding, spatial hearing, and sound quality. Participants answered questions (14 to 18 per domain) using a 10-point Likert scale in which 10 indicated they could hear/understand perfectly in the communication scenario and 0 indicated they could hear/understand not at all.

### dMRI

dMRI data for 10 participants with NF1 and 10 matched control participants were acquired using a Siemens Magnetom Prisma 3T MRI scanner with a 32-channel head coil. The following scanning parameters were used: repetition time, 3300 milliseconds; echo time, 71 milliseconds; 2.5 mm isotropic voxels; field of view, 260 × 260 mm; matrix size, 96 × 96, and acceleration factor, 2. Sixty-four diffusion weighted images (degree of diffusion weighted [b], 2800 s/mm^2^) and 4 non–diffusion weighted images (b = 0 s/mm^2^) were acquired with a total acquisition time of approximately 10 minutes. Processing of the dMRI data followed previously outlined steps.^21^ Specifically, a fixel-based analysis, yielding metrics of apparent fiber density (FD) and fiber-bundle cross-section (FC), was performed on auditory brainstem tracts. This was achieved using probabilistic tractography to delineate the tracts running between the vestibulocochlear nerve and inferior colliculus bilaterally on the population template image.^22^ The generated tractogram was converted to a fixel mask to allow fixel-based analysis to be performed in a tract-specific manner. The apparent FD metric is proportional to the intra-axonal volume of axons within a voxel and therefore is sensitive to changes in white matter microstructure. Comparatively, FC is a measure of white matter macrostructure and can be used to determine changes within the cross-sectional distribution of axons within a fiber bundle.^23^

### Statistical Analysis

Data were analyzed with the MINITAB version 19 package. All assumptions for parametric analyses were met. Normality of data distribution was assessed using Shapiro-Wilk and Kolmogorov-Smirnov tests. Group differences for dimensional outcomes were explored using regression-based models with covariate adjustments for age and hearing level. Unadjusted comparisons were performed using paired *t* tests. Group differences for categorical outcomes were examined using χ^2^ analyses and odds ratios (ORs).

Diffusion MRI data were processed and analyzed using the MRtrix3Tissue version 5.2.8 software package.^24^ Preprocessing steps included denoising,^25^ removal of Gibbs-ringing artifact,^26^ and eddy-current distortion, motion, and EPI-susceptibility distortion correction.^27,28^ Subsequently, diffusion directions were modeled using single-shell 3-tissue constrained spherical deconvolution.^29^ Nonparametric permutation testing over 5000 permutations was used to determine which fixels (specific fiber population in a voxel) were significantly different between NF1 and control groups using connectivity-based fixel enhancement.^30^ Two-tailed familywise error–corrected *P* values were assigned to each fixel, and significant fixels (*P* < .05) were visualized according to a color gradient.

## Results

A total of 44 participants (18 [41%] female individuals) and 44 control participants (18 [41%] female individuals) were included. The mean (SD) age in the NF1 group was 16.9 (10.7) years and, in the control group, 17.2 (10.2) years.

### Sound Detection Thresholds

Overall, 42 of 44 participants (96%) with NF1 presented with normal sound detection (4-frequency average hearing level, <20 dBHL) (Table 1). One adult showed mild high-frequency (sensory) hearing loss consistent with a history of noise exposure, and 1 child presented with mild conductive loss and evidence of middle ear disease.

### Auditory Electrophysiological Responses

**Figure 1.**
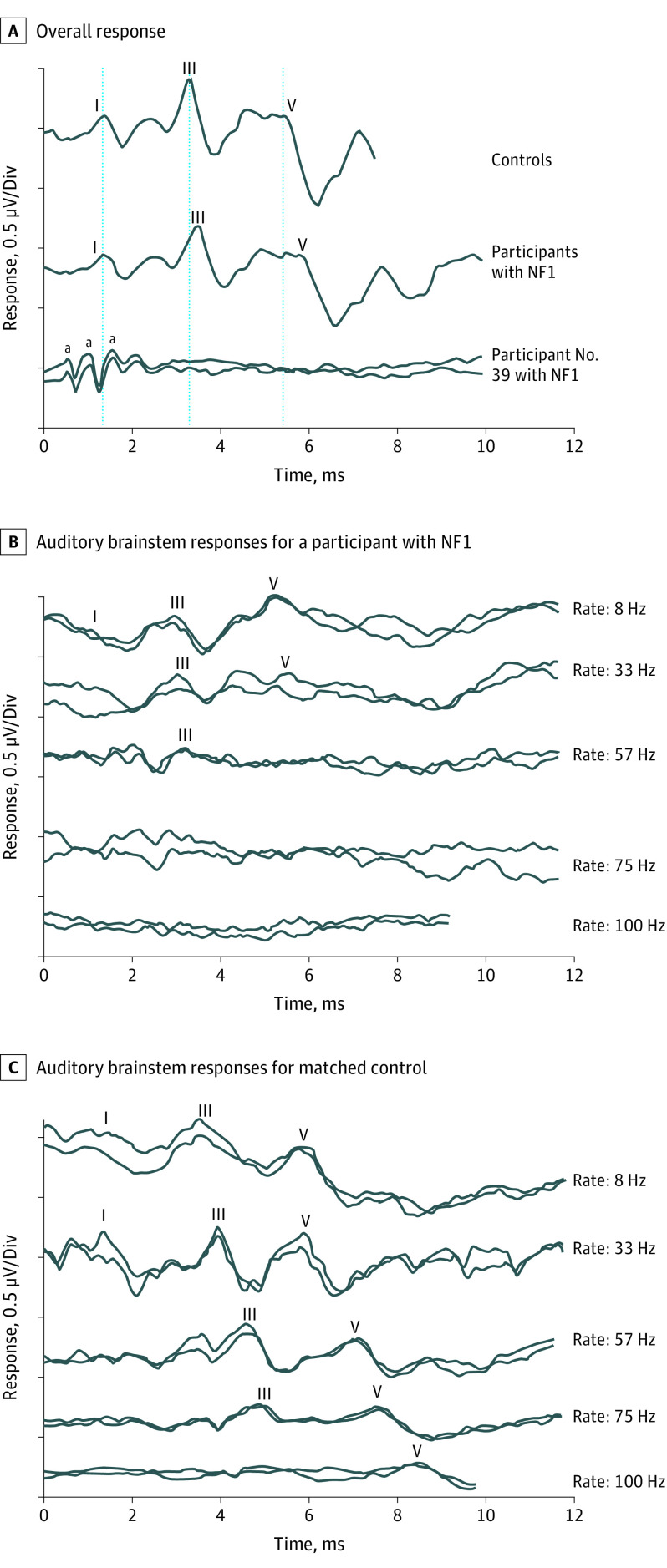
Average Auditory Brainstem Response Waveforms for Neurofibromatosis 1 (NF1) and Control Cohorts A, The bottom tracings show present cochlear microphonic responses and absent auditory neural potentials for participant 39 in the NF1 cohort. (The cochlear microphonic is a scalp-recorded electrical potential generated by the polarization and depolarization of cochlear hair cells.) B and C, Auditory brainstem responses for a participant with NF1 (B) and her matched control participant (C) to acoustic click stimuli at presentation rates ranging from 8 Hz to 100 Hz. ^a^Positive peaks in the cochlear microphonic waveform.

Repeatable ABRs were obtained for 43 participants with NF1 (97.7%). One individual showed absent potentials despite normal sound detection and present preneural responses from the cochlear hair cells (cochlear microphonic potentials) (Figure 1A).

Significance testing for group differences on all continuous audiological outcomes were consistent across adjusted and unadjusted analyses. Neural conduction velocity was lower in participants with NF1 compared with control participants (Table 2). While there were no group differences for ABR wave I latency, individuals with NF1 demonstrated significantly longer latencies for waves III and V (Table 2). Wave I to III interpeak latencies were equivalent between groups, whereas wave III to V latencies were longer in participants with NF1 compared with control participants. Ten of 43 participants (23%) with NF1 and repeatable ABRs showed interpeak latencies (wave III-V) outside published reference ranges.^13^ This proportion was significantly greater than for the control group, in which only 1 participant (2%) had abnormal interpeak latencies (OR, 13.03; 95% CI, 1.59-106.95; *P* = .003). ABR amplitude was also significantly different in the NF1 and control groups. While wave I showed no group difference, wave V peak-to-peak amplitude was significantly lower in individuals with NF1 (Table 2).

**Table 2.** Auditory Brainstem Response, Perception, and Self-reported Hearing Disability Findings for Participants With NF1 and Matched Control Participants

Outcome	Mean (SD)			95% CI for paired difference	*P* value	
	Participants with NF1	Control participants	Difference		Unadjusted	Adjusted^a^
**Auditory brainstem response**						
Wave I latency, ms	1.50 (0.12)	1.45 (0.13)	0.05 (0.17)	−0.00 to 0.10	.06	.08
Wave III latency, ms	3.75 (0.17)	3.64 (0.15)	0.11 (0.21)	0.05 to 0.18	.001	.004
Wave V latency, ms	5.77 (0.31)	5.41 (0.16)	0.36 (0.35)	0.25 to 0.47	<.001	<.001
I-III Interpeak latency, ms	2.27 (0.20)	2.19 (0.16)	0.07 (0.27)	−0.00 to 0.16	.07	.06
III-V Interpeak latency, ms	2.02 (0.26)	1.78 (0.13)	0.24 (0.29)	0.16 to 0.33	<.001	<.001
Wave I amplitude, μV	0.34 (0.12)	0.36 (0.12)	−0.02 (0.14)	−0.09 to 0.04	.44	.32
Wave V amplitude, μV	0.59 (0.23)	0.69 (0.22)	−0.11 (0.31)	−0.20 to −0.01	.03	.02
Maximum rate, Hz^b^	81.7 (26.5)	98.9 (5.3)	−17.1 (26.9)	−25.3 to −9.0	<.001	<.001
**Temporal processing**						
10-Hz AM, dB	−16.3 (4.9)	−18.1 (3.8)	1.7 (6.0)	−0.17 to 3.64	.07	.16
150-HZ AM, dB	−13.8 (5.6)	−17.1 (2.5)	3.4 (6.1)	1.33 to 5.37	.002	.004
**Binaural speech perception in noise**						
DV90, dB	−13.2 (3.8)	−15.8 (2.8)	2.6 (4.0)	1.40 to 3.82	<.001	.002
SV90, dB	−11.5 (3.9)	−14.1 (2.9)	2.6 (3.9)	1.42 to 3.82	<.001	.002
DV0, dB	−5.0 (3.4)	−5.9 (2.5)	0.9 (3.9)	−0.27 to 2.09	.15	.18
SV0, dB	−1.1 (1.9)	−1.3 (1.7)	0.2 (2.2)	−0.46 to 0.86	.89	.69
**Hearing disability** ^c^						
Speech	7.3 (1.6)	8.3 (0.9)	−1.0 (1.8)	−1.61 to −0.47	<.001	<.001
Spatial	6.8 (2.2)	8.0 (1.4)	−1.1 (2.7)	−2.00 to −0.28	.01	.009
Quality	8.1 (1.6)	8.4 (1.1)	−0.2 (2.1)	−0.91 to 0.44	.49	.37

Abbreviations: AM, amplitude modulation; DV0, different voice, same direction; DV90, different voice separated by 90°; NF1, neurofibromatosis type 1; SV0, same voice, same direction; SV90, same voice separated by 90°.

^a^ Adjusted for age and hearing level.

^b^ Maximum rate is the highest stimulus presentation rate (in hertz) at which an auditory brainstem response could be identified.

^c^ Hearing disability ratings are based on a 10-point rating scale.

Auditory brainstem potentials for individuals with NF1 were abnormally sensitive to increases in stimulus presentation rate (Figure 1B and C). The maximum rate at which a repeatable waveform could be identified was significantly lower in participants with NF1 than in control participants (Table 2).

### Auditory Temporal Processing

Temporal resolution results were abnormal in individuals with NF1. While there was no group difference for low-rate (ie, 10-Hz) modulation detection thresholds (Table 2), participants with NF1 required a greater depth of modulation to identify the amplitude variation for high-rate (ie, 150-Hz) amplitude modulations.

### Speech Perception

Fourteen of 44 participants (32%) with NF1 showed clinically abnormal binaural speech perception, with SRT values more than 2 SDs less than age-based means for the DV90° and/or SV90° listening conditions.^19^ This proportion was significantly higher than the control group, in which only 1 individual (2%) scored outside the reference range (OR, 20.07; 95% CI, 2.50-160.89; *P* < .001).

Analyses for the 2 listening conditions in which target sentences and noise were presented from different directions showed significant group differences (Table 2). Mean SRT for the NF1 group was 2.6 dB poorer than for the control group in both conditions. In contrast, there were no group differences for conditions in which speech and noise emanated from the same direction.

### Self-reported Hearing Disability

Individuals with NF1 reported higher degrees of everyday speech understanding and communication difficulty than their matched peers (Table 2). Both the SSQ–speech perception and spatial hearing domains were lower for patients with NF1 than those in the control groups. Ratings for sound quality showed no group difference.

### Associations Between Auditory Measures

Auditory neural function in the late brainstem was reflected in behavioral hearing ability. Pearson correlations were used to investigate associations between neural conduction efficiency (ABR wave III-V), temporal processing (detection with amplitude modulation of 150 Hz), binaural speech perception (DV90 condition), and hearing disability (SSQ–speech domain). ABR interpeak latency was correlated with each of the other measures, such that individuals with the slowest auditory neural conduction velocities also presented with the poorest temporal resolution, greatest speech perception difficulty, and highest degree of speech/communication challenges in everyday listening (Table 3).

**Table 3.** Pairwise Pearson *r* Correlations Showing the Associations Between Selected Auditory Measures

Measure 1	Measure 2	Correlation (95% CI)	*P* value
ABR III-V	AM 150Hz	0.316 (0.10 to 0.50)	.004
ABR III-V	DV90	0.317 (0.11 to 0.49)	.003
ABR III-V	SSQ Speech	−0.229 (−0.42 to −0.01)	.04
AM 150Hz	DV90	0.369 (0.16 to 0.54)	.001
AM 150Hz	SSQ Speech	−0.101 (−0.32 to 0.13)	.38
DV90	SSQ Speech	−0.231 (−0.42 to −0.02)	.03

Abbreviations: ABR, auditory brainstem responses; AM, amplitude modulation; DV90, different voices spatially separated by 90°; SSQ, Speech, Spatial, and Qualities of Hearing Scale.

### Fixel-Based Analysis of dMRI

**Figure 2.**
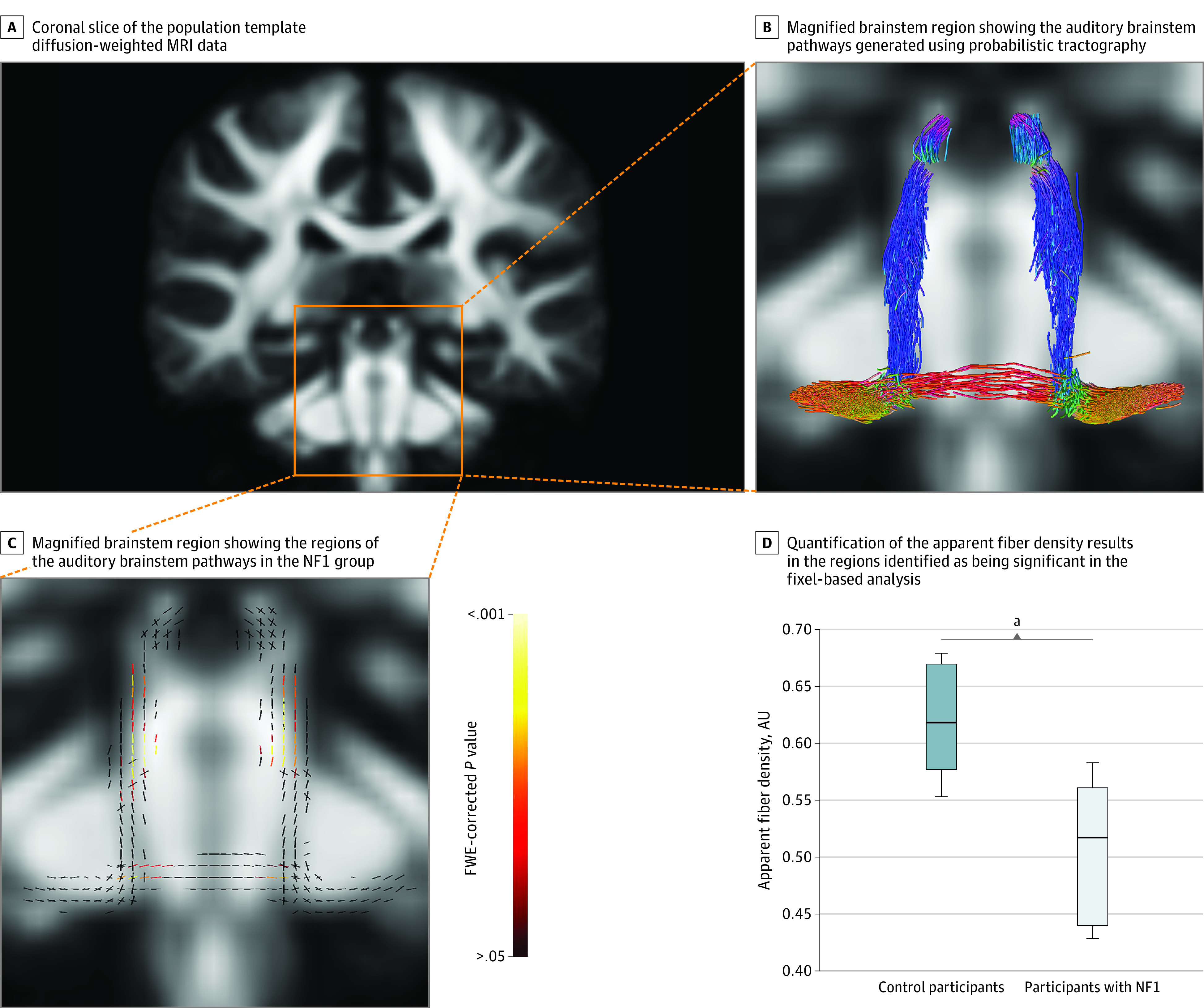
Fixel-Based Analysis Results A, Coronal slice of the population template diffusion-weighted magnetic resonance imaging (MRI) data, with the box indicating the brainstem where the analysis was performed. B, Magnified brainstem region showing the auditory brainstem pathways (generated using probabilistic tractography)^22^ in which apparent fiber density was compared between the neurofibromatosis type 1 (NF1) group and control group.^21^ The different colors of the tracts in this image correspond to tract orientation and adhere to the directionally encoded color convention: anterior-posterior is green, superior-inferior is blue, and left-right is red. C, Magnified brainstem region showing the regions of the auditory brainstem pathways in the NF1 group that exhibited significantly lower apparent fiber density (ie, presence of neural degeneration) compared with the control group. This image shows the auditory pathways as fixels (individual fiber populations in a voxel), which are color-coded based on familywise error (FWE)–corrected *P* values. D, Quantification of the apparent fiber density results in the regions identified as being significant in the fixel-based analysis (C). Values are represented in arbitrary units (AUs). These boxplots present the median (center line), the interquartile range (shaded area of the box), and whiskers (range) of the data. ^a^*P* < .001.

The fixel-based analysis of the auditory brainstem pathways (Figure 2A and B) showed specific regions in which the apparent FD metric was significantly lower in the NF1 group compared with the control group (median [IQR] AFD, 0.517 [0.440-0.561] arbitrary units vs 0.619 [0.578-0.670] arbitrary units; *P* < .001) (Figure 2C and D). These regions were found to occur only in the ascending auditory tracts between areas corresponding to the cochlear nuclei and inferior colliculi. There were no significant differences between the NF1 group and the control group for the FC metric.

## Discussion

This study revealed significant auditory pathway abnormalities and perceptual deficits in our population of patients with NF1. Despite normal or near-normal sound detection thresholds, approximately 1 in 3 patients with NF1 presented with functional hearing abnormalities severe enough to impede developmental progress in children and restrict everyday communication in older participants.

Results of auditory brainstem testing were abnormal in many participants with NF1. One individual displayed the classic auditory neuropathy pattern, presenting with absent vestibulocochlear nerve/brainstem potentials in conjunction with normal sound detection and normal responses from the cochlear hair cells.^11^ Furthermore, 23% of the cohort had clinically abnormal neural conduction, especially in the late brainstem region (between cochlear nucleus and lateral lemniscus) and reduced response amplitude. This ABR pattern resembles that found in patients with central demyelinating processes, such as multiple sclerosis.^31^ Demyelination increases membrane capacitance and decreases resistance, leading to delayed and/or diminished excitation and reductions in the velocity and synchrony of action potential propagation.^31,32,33^ The findings are also consistent with those seen in patients with pontine angle tumors, such as vestibular schwannomas and meningiomas, where neural compression results in prolongation of wave I to V conduction and axonal loss results in ABR amplitude reduction.^33^

dMRI data from previous studies in individuals with NF1 have identified generalized white matter disorganization consistent with abnormal connectivity and slowed processing within neural networks.^34,35^ Analysis of dMRI data in our study extends these findings, providing a microstructural and macrostructural analysis of the white matter pathways of the auditory brainstem. The fixel-based analysis showed significantly lower apparent FD within the ascending auditory tracts of participants with NF1. Specifically, the regions involved corresponded to the tracts running between the cochlear nucleus and inferior colliculus bilaterally (ie, late brainstem). This result can be attributed to either a loss of axons within these regions or abnormal or delayed axonal development resulting in reduced axon diameter in participants with NF1. Within the context of the ABR results, however, axonal loss is the more likely explanation for the observed reduction in evoked response amplitude. Moreover, secondary demyelination may occur as a consequence of axonopathy and could therefore account for the prolonged neural conduction times recorded in participants with NF1.^36^

Auditory brainstem response testing also showed an increased vulnerability to high-rate stimuli, suggesting that the auditory systems of participants with NF1 were more easily stressed than matched control participants. Comparable results have been reported for other neuropathologies^37^ and in experimental studies of induced axonal and demyelinating disorders.^38^

Consistent with the rate effect observed in the evoked potential recordings, participants with NF1 demonstrated an impaired ability to perceive rapidly occurring changes in acoustic stimuli. While detection of amplitude variations over a slow (ie, 100-millisecond) time course was normal, identification of rapid-amplitude (ie, 6.7-millisecond) modulation was significantly impaired, suggesting that auditory neural pathways were less able to encode brief acoustic fluctuations. Temporal resolution deficits are a cardinal feature of auditory neural pathology and have been reported in other patient populations with disrupted central auditory function, including axonal and demyelinating neuropathy,^11^ autism,^39^ and diabetic neuropathy.^40^

The major functional consequence of disrupted temporal processing is impaired speech discrimination. In optimal (ie, quiet) conditions, affected listeners struggle to identify timing-based phonemic cues, such as consonant voicing or vowel duration.^13^ With background noise, they show extreme masking effects, primarily as a result of impaired binaural processing. Distortion of neural firing patterns from each ear means that signals cannot be effectively combined in the central auditory pathways.^18^ As a result, the listener is less able to localize sound sources and differentiate target speech and background noise when they emanate from different directions.^41^ This process, known as spatial streaming, affords a release of at least 10 dB from the masking effects of noise in individuals with normal binaural processing^19^ and is consistently lower in individuals with auditory neural abnormality.^10^

Many participants with NF1 demonstrated impaired speech perception in noise as a result of impaired spatial streaming. Fourteen of 44 (32%) patients showed clinically abnormal perception for listening conditions where speech and noise were spatially separated by 90°. Speech reception thresholds in these cases were, on average, 2.6 dB lower among individuals with NF1 than among the control group, suggesting that for many patients with NF1, everyday listening environments are experienced as significantly noisier than for control participants. This degree of deficit is functionally significant and known to affect academic outcomes,^42^ cognitive function, and physiologic stress levels.^43^

Consistent with the perceptual deficits measured in the laboratory, individuals with NF1 reported greater everyday listening and communication difficulties than matched control participants. While they felt sounds were generally clear and undistorted, both child and adult participants reported challenges with sound localization and speech understanding across a range of scenarios, including group communication and classroom listening.

The degree of perceptual deficit among participants with NF1 was such that if their auditory dysfunction had been associated with cochlear hearing loss, they would have met candidacy criteria for hearing aids or cochlear implants.^44^ Unfortunately, these devices are not appropriate for patients with NF1, as their difficulties are not the result of impaired sound detection but a distorted neural representation of sounds. Hearing aids make sounds louder but do not make them clearer.^10^ Disrupted neural activity in the late brainstem appeared to be the source of this abnormality, and individuals with the greatest degree of ABR abnormality presented with the most impaired auditory processing and functional hearing. dMRI findings indicated that the pathophysiological process underpinning these hearing difficulties is likely to be axonopathy occurring within the ascending auditory tracts of the brainstem. Abnormal or delayed axonal development resulting in reduced axon diameter, however, cannot be excluded.

The major hearing consequence identified in this study was an impaired capacity to discriminate speech in the presence of background noise. Clinically, speech understanding and communication is improved in individuals with all forms of hearing loss by increasing the level of the speaker’s voice relative to the competing noise. This may be achieved by configuring the environment to minimize noise or by increasing the level of the speech signal. In the latter case, remote-microphone listening systems improve the signal-to-noise ratio (by ≥10 dB) by recording the speaker’s voice near the mouth and transmitting the signal directly to the listener’s ear. Such devices have proven useful in other populations with auditory neural deficit—improving hearing, communication, and academic outcomes—and may be beneficial in individuals with NF1.^39,45^

### Limitations

This study has limitations. While the focus of this study was pathophysiological changes occurring in the auditory brainstem of patients with NF1, auditory neural pathology may also involve more central regions. Future studies might include electrophysiologic and dMRI assessments of the auditory cortices. Furthermore, while we did not image all participants for brain tumors at screening, we excluded participants with symptomatic brain pathology, so it is unlikely that brain tumors were associated with the audiological deficits. Additionally, we acknowledge the subsample of participants that completed neuroimaging was small; however, we believe it is representative of the broader NF1 population and is consistent with previous reports of dMRIs showing white matter abnormalities in patients with the condition.^34^

## Conclusions

In this study, many patients with NF1 experienced auditory processing deficits indicating a new clinical and neurobiological feature of the condition. More than 30% demonstrated clinically abnormal speech perception. The findings suggest that auditory evaluation should be part of the management regime for every patient with NF1 and that the test protocol should include electrophysiological and speech perception (in noise) assessment. Standard audiometry is inadequate for this group, as sound detection can be normal in individuals with severe functional hearing limitations.
